# Pt-TiO_2_ Systems for Enhanced Hydrogen Production from Glycerol: Direct vs Sequential Incorporation Through Photodeposition

**DOI:** 10.3390/ma17205109

**Published:** 2024-10-19

**Authors:** Ana M. Carozo, Francisco J. López-Tenllado, M. Carmen Herrera-Beurnio, Jesús Hidalgo-Carrillo, Juan Martín-Gómez, Rafael Estevez, Alejandro Ariza-Pérez, Francisco J. Urbano, Alberto Marinas

**Affiliations:** Departamento de Química Orgánica, Instituto Químico para la Energía y el Medioambiente (IQUEMA), Universidad de Córdoba, E-14071 Córdoba, Spain; anamcarozo@gmail.com (A.M.C.); b42lotef@uco.es (F.J.L.-T.); jesus.hidalgo@uco.es (J.H.-C.); q92magoj@uco.es (J.M.-G.); q72estor@uco.es (R.E.); q82arpea@uco.es (A.A.-P.); qo1urnaf@uco.es (F.J.U.)

**Keywords:** photocatalysis, TiO_2_, photodeposition, metal loading, glycerol photoreforming, hydrogen production

## Abstract

Pt-TiO_2_ systems are the most widely used photocatalysts in the production of green hydrogen from glycerol photoreforming. To incorporate metals on the surface of materials, photodeposition is the most used method because it employs mild conditions. However, despite its use, there are some parameters that have not been deeply studied, such as the appropriate metal loading and the method itself, to obtain a better dispersion of Pt. In this work, six Pt-TiO_2_ catalysts were synthesized by a classical photodeposition method employing UV radiation. The studied Pt wt.% range was 0.15–0.60 wt.%, being incorporated in one step or in subsequent ones. HRTEM analyses showed that both methods allowed a homogeneous distribution of Pt, and in both, the particle size was around 2.3–3.6 nm, increasing with metal loading. The photocatalytic activity of materials was tested in glycerol photoreforming under UV radiation, and the 0.45 wt.% Pt-containing solid that had been synthesized in one step was the one that allowed the highest hydrogen production. This might suggest that around 0.40% is the appropriate metal loading for hydrogen production under these conditions and that incorporating the desired metal percentage in one step is the most efficient method in terms of energy and time savings.

## 1. Introduction

It is intended that hydrogen will play a leading role in the context of the energy transition from fossil fuels to renewable energy. This gas has a high calorific value of 122 kJ·g^−1^ and only produces water vapor in its combustion, thus reducing CO_2_ emissions, which are involved in the greenhouse effect and global warming [1]. However, hydrogen in its molecular form is not very abundant in nature. Although some H_2_ wells have recently been found [2], hydrogen is mainly bonded to other elements, so to use it as an energy source, it must be produced. Hydrogen production through photocatalytic biomass reforming is an interesting option as this method only requires light, a photocatalyst, water, and an organic compound derived from biomass that acts as an electron donor [3]. In this sense, photocatalytic reforming of biomass is a sustainable process: on the one hand, as CO_2_ produced from these reactions is obtained from biomass, it belongs to the carbon cycle and has no influence on the greenhouse effect [4]; on the other hand, this procedure allows valorizing biomass, which favors a circular economy. A sacrificial agent that has gained importance is glycerol, since it is the main by-product in the production of biodiesel. From producing 10 kg of biodiesel, 1 kg of glycerol is obtained. Thus, its valorization into chemicals, fuels, or value-added products can make full use of the surplus of this organic compound [5].

The overall glycerol photoreforming reaction is as follows:

(1) $$C_{3}H_{8}O_{3}+3\;H_{2}O\;\to \;7\;H_{2}\;+\;3\;{CO}_{2}$$

In this reaction, 7 moles of hydrogen and 3 moles of carbon dioxide are obtained. Therefore, complete photoreforming would mean a H_2_/CO_2_ ratio of 2.33. However, generally, this stoichiometric relationship is not fully reached, and the ratio tends to be over that value as oxidized intermediates such as organic acids, ketones, or aldehydes accumulate in the reaction medium [6].

The most commonly used photocatalyst is TiO_2_ [7] due to its low cost, high availability, photocorrosion, resistance, biocompatibility, high chemical stability, etc. However, it has two main disadvantages: (i) it has a band gap of 3.0–3.2 eV, which limits its absorption to the UV range (c.a. 5% of solar radiation), and (ii) a quick electron–hole pair recombination, as approximately 90% of photoexcited electrons are recombined within 10 ns after excitement [8,9]. To deal with these two main disadvantages of TiO_2_, there are different approaches. For example, there is the creation of heterojunctions between TiO_2_ and another semiconductor with an absorption capacity in the visible spectrum. In this way, a material that absorbs in this range of the spectrum is generated, and it also has a slower electron–hole pair recombination, due to the electron transfer between the conduction or valence bands of the semiconductors that form the heterojunction. Another common practice is doping TiO_2_ with metallic or non-metallic nanoparticles, which implies the incorporation of these nanoparticles into the structure of the oxide, creating defects in its bandgap or narrowing it to allow its visible light range absorption [10]. Among metal doping, noble metals such as Pt, Pd, Ru, or Au stand out [11]. In addition, incorporating metals into the surface of TiO_2_ is a widely used technique in which these charge carriers reduce the recombination of the electron–hole pair due to the electron transfer between the conduction band of the semiconductor and the Fermi level of the metal [12]. Again, among the metals that are incorporated into the surface of the materials, noble metals are the most widely used, especially platinum, because it has demonstrated a substantial improvement in hydrogen production [13,14,15,16]. Nevertheless, given their high cost, their content should be carefully optimized. There are also attempts at replacing noble by non-noble metals such as copper or nickel [17,18].

There are different methods of incorporating metals into the surface of TiO_2_. One of them is impregnation, where the precursor solution is added over the catalyst, taking the pore volume of the solid into account to add it in excess or in the exact amount of that volume to allow the accurate incorporation of the metal into the catalyst. Then, the solid is calcined and reduced to obtain the zero-oxidation state. Another technique is electrodeposition, which allows metal incorporation after the reduction of an electrolyte to generate a solid in the cathode of an electrochemical cell. During deposition-precipitation, metals are incorporated after the precipitation of the precursor of the desired metal due to the pH of the medium or some precipitation agent. Colloidal synthesis is carried out through several steps in which the catalyst is synthesized in a solvent using a surfactant as a protecting agent, to incorporate the colloid and reduce the precursor with NaBH_4_ or other chemical reagent [19,20,21].

Apart from these, there is another method that stands out, due to the mild conditions that are needed to carry it out, and it is photodeposition. In this procedure, the semiconductor is added together with a sacrificial agent that contains the metal precursor. This slurry is illuminated for several hours allowing the incorporation of metal nanoparticles in their elemental state on the surface of the photocatalyst. The use of a sacrificial agent is again essential as its oxidation allows the reduction of the metal. Among the sacrificial agents, methanol stands out, as after being illuminated, it produces methoxy radicals that might enhance the efficiency of the reaction by inducing the incorporation of the metal nanoparticle on the surface of the semiconductor [22]. Methanol also allows a homogeneous distribution of the metal; however, the proportion of this organic compound must be controlled, as it has been shown that high concentrations of methanol in the reaction medium can induce the agglomeration of nanoparticles and, therefore, reduce the photocatalytic efficiency of the material [22,23].

Although photodeposition is a common process, there are still some parameters that have not been studied in depth, such as the optimum metal loading. This variable creates some controversy, as the metal loading values found in the literature are very diverse [24,25,26]. Also, although it is known that high concentrations of the sacrificial agent might negatively influence the homogeneity of the particle distribution, the synthetic method itself should be carefully examined, to allow a homogeneous distribution of metal nanoparticles on the surface of TiO_2_.

In this work, some Pt/TiO_2_ systems will be synthesized using two different photodeposition procedures. In the first one, a metal precursor will be added in order to obtain the desired metal loading on the semiconductor (direct method); in the second one, sequential photodeposition reactions will be carried out to increase the metal loading by adding a constant volume of metal precursor (sequential method). With this, it will be possible to observe whether the presence of platinum nanoparticles in the semiconductor allows the metal to act as a nucleation point in a consecutive photodeposition process, easing the incorporation of the metal. TiO_2_ systems with platinum will be synthesized using P25 as the semiconductor, commercial titania, and chloroplatinic acid (H_2_PtCl_6_) as the metal precursor. Therefore, the aim of this study is to optimize Pt metal loading along with the photodeposition procedure so as to obtain high-efficiency photocatalysts for hydrogen production through glycerol photoreforming reactions.

## 2. Materials and Methods

### 2.1. Synthesis of the Photocatalysts

The Pt/TiO_2_ systems used in this work were synthesized using commercial TiO_2_ (P25 Degussa Evonik), on which platinum was incorporated through a typical photodeposition method using H_2_PtCl_6_ as the precursor. Pt was incorporated through two experimental procedures: directly, by adding the appropriate H_2_PtCl_6_ volume in order to incorporate the desired metal loading into P25, or sequentially, increasing the metal loading on each photodeposition reaction and keeping constant the volume of the precursor used in each step. The chosen Pt nominal contents for metal loading were 0.15 wt.%, 0.30 wt.%, 0.45 wt.%, and 0.60 wt.%. The systems were denoted as X(d)-Pt/TiO_2_ and X(s)-Pt/TiO_2_, ‘*X*’ being the nominal content of Pt in the sample, and ‘d’ and ‘s’ being direct or sequential, respectively, to refer to the way Pt was incorporated into TiO_2_.

To carry out the direct photodeposition, 65 mL of a 10% (*v*/*v*) aqueous solution of methanol was added into a 190 cm^3^ cylindrical reactor. A total of 0.5 g of P25 was incorporated into the solution, followed by the appropriate H_2_PtCl_6_ volume. The reaction was irradiated for 5 h using a 125 W Hg lamp (564 mW·cm^−2^), keeping the temperature constant at 25 °C and the inert atmosphere under an Ar flow of 20 mL·min^−1^. After 5 h, solids were filtered, washed with distilled water, and dried at 110 °C overnight. In the ‘direct’ series, 4 systems were obtained: 0.15(d)-Pt/TiO_2_, 0.30(d)-Pt/TiO_2_, 0.45(d)-Pt/TiO_2_, and 0.60(d)-Pt/TiO_2_. For the sequential one, the photodeposition experimental conditions were the same, but this time, 0.15(d)-Pt/TiO_2_ was added instead of P25. H_2_PtCl_6_ was incorporated into the solution medium so as to obtain the 0.30(s)-Pt/TiO_2_ system. The same procedure was again carried out using 0.30(s)-Pt/TiO_2_ to generate the 0.45(s)-Pt/TiO_2_ catalyst. The 0.60 wt.% semiconductor was not synthesized through the sequential method, as some preliminary glycerol photoreforming reactions showed that the optimum Pt content for efficient hydrogen production was not around this value. The solids 0.30(s)-Pt/TiO_2_ and 0.45(s)-Pt/TiO_2_ were obtained after filtering, washing with distilled water, and drying at 110 °C overnight.

### 2.2. Characterization of the Semiconductors

The platinum content was determined by using Inductively Coupled Plasma Mass Spectrometry (ICP-MS) on a Perkin Elmer Nexion350X spectrometer (Waltham, MA, USA) that was equipped with argon plasma ionization, ion-detecting quadrupole detection, and a sample-introducing system. The spectrometer was located at the Central Service for Research Support (SCAI) of the University of Córdoba.

X-ray photoelectron spectra (XPS) were also carried out at the SCAI of the University of Córdoba. This technique was used to determine the surface Pt content and oxidation states of the metal. Catalysts were compacted on pellets and outgassed to a pressure below 6·10^−9^ mbar at room temperature. The spectrometer was Phoibos 150 MCD (Specs, Berlin, Germany), and it operated with a AlKα (hν = 1486.6 eV) X-ray source at 400 W using as reference C1s (284.8 eV). As Ti3s has a satellite in the Pt4f region around 73.5–73.7 eV [27]; the signal that was obtained for this satellite was subtracted from the signal of Pt4f.

High-Resolution Transmission Electron Microscopy (HRTEM) was carried out on a ThermoScientific Talos F200i (Waltham, MA, USA) microscope operating at 200 kV at the SCAI of the University of Córdoba. A total of 100 platinum particles were counted out from HRTEM images to determine the Pt particle size distribution and the agglomeration of the metal using ImageJ software (version 1.50i).

Diffuse reflectance UV-Vis spectroscopy was carried out on a PerkinElmer Lambda 650 S UV/Vis Spectrometer (Waltham, MA, USA) equipped with a 60 mm integration sphere and utilizing BaSO_4_ as a reference. Analyses were carried out in the wavelength range of 250–800 nm. The bandgap value of the semiconductors was calculated by the plot of the modified Kubelka–Munk function (αhν^1/2^, as TiO_2_ has an indirect transition bandgap) versus the energy of the light absorbed by the sample (eV). Also, the absorbance of samples was obtained through this technique.

X-ray Diffraction (XRD) was carried out on a Bruker D8 Discover (Bruker Española S.A., Madrid, Spain) with monochromatic CuKα1 radiation (λ = 1.54 Å) with a scan speed of 2.31 °2ϴ·min^−1^ over an angular range of 5–80°. The equipment was located at the Chemical Institute for Energy and Environment (IQUEMA) of the University of Córdoba. XRD analyses were used to determine the crystallographic phases of TiO_2_ and platinum.

### 2.3. Glycerol Photoreforming Reactions

Glycerol photoreforming was carried out on a Penn PhD m2 photoreactor using LED light (λ = 365 nm, 213 mW·cm^−2^). A total of 1 g·L^−1^ of suspensions of catalysts on a 10% (*v*/*v*) aqueous solution of glycerol was added to a 20 mL Pyrex cylindrical tube. Solids were irradiated for 6 h, with the previously mentioned photoreactor maintaining constant stirring and an inert atmosphere, as a 4 mL·min^−1^ Ar flow was bubbled throughout the whole reaction. The outlet gas composition was on-line analyzed by gas chromatography with a thermal conductivity detector (TCD) on an Agilent Technologies 7890A gas chromatograph (Agilent Technologies, Santa Clara, CA, USA) equipped with a Supelco Carboxen^TM^ 1010 Plot Column.

## 3. Results and Discussion

### 3.1. Characterization of the Photocatalysts

Table 1 summarizes the results obtained from the characterization of the solids. Regarding the characterization of platinum, ICP-MS results show that the experimental Pt content was in the 0.17–0.52 wt. % range, being close to the nominal value for each sample. As in all cases, the metal incorporation had a yield of over 85%, and it can be concluded that photodeposition is an efficient experimental procedure to incorporate Pt into titania.

Surface analyses were performed by XPS. These results provide evidence that platinum is mainly found as Pt^0^ in all samples, and it is also possible to find Pt^2+^ in all of them. The high platinum zero content found in the samples is expected in the photodeposition technique, as the use of UV radiation allows the Pt^4+^ present in chloroplatinic acid to be reduced to the elemental state due to the high energy of this type of radiation. The metal was not identified in the 0.15(d)-Pt/TiO_2_ semiconductor due to the low platinum content that was incorporated through photodeposition [28]. The low percentages of Pt^4+^ identified in the 0.45(d)-Pt/TiO_2_, 0.45(s)-Pt/TiO_2_, and 0.60(d)-Pt/TiO_2_ systems might be due to the oxidation of the surface that the catalyst might have undergone from its synthesis until the analysis was carried out. XPS Pt4f spectra of all catalysts are shown in Figure S1.

Regarding Pt^0^ binding energies, all systems show a binding energy for Pt4f7/2 of 70.7–70.8 eV, which is consistent with results shown in XPS analyses for Pt/TiO_2_ samples in the literature [29,30]. The surface platinum content determined by XPS is slightly higher than the bulk content (determined by ICP-MS). This fact agrees with the employed synthesis procedure, as platinum has been photodeposited on the surface of TiO_2_.

**Figure 1:**
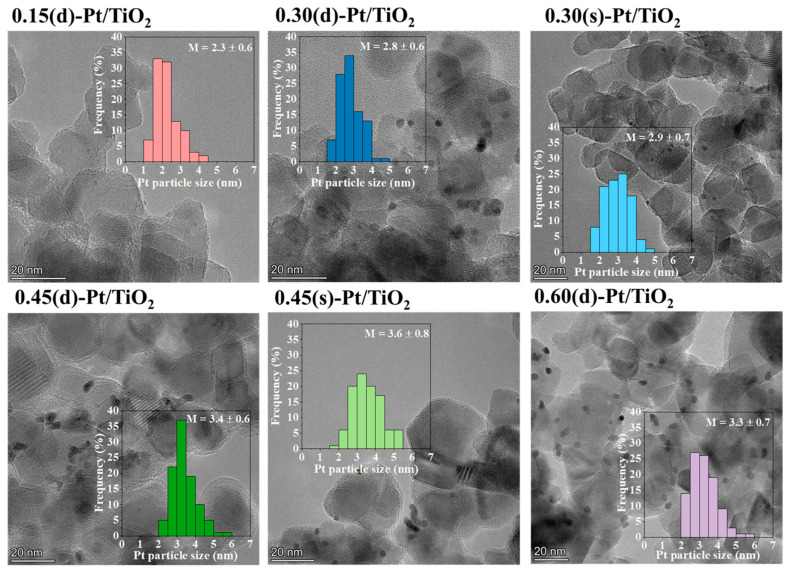
HRTEM micrographs of semiconductors synthesized in this work. Platinum particle size histograms along with the average particle sizes are also depicted.

HRTEM micrographs are shown in Figure 1 along with Pt particle size histograms. It can be observed for photocatalysts synthesized in several photodeposition reactions (sequential series) that platinum is slightly more agglomerated or clustered, whereas a more homogeneous distribution is visible on TiO_2_ systems synthesized in a single step (direct series). This might be caused by the fact that the Pt that was already present in the solids 0.15(d)-Pt/TiO_2_ and 0.30(s)-Pt/TiO_2_, used as supports for consecutive photodeposition reactions, may act as a nucleation point, causing platinum clusters.

However, in all samples, the Pt particle size ranges between 2.3 and 3.6 nm, with a noticeable increase as the platinum loading increases from 0.15 wt.% to 0.60 wt.% (2.3 nm at 0.15 wt.%, 2.8 nm at 0.30 wt.%, 3.4 nm at 0.45 wt.%, and 3.3 nm at 0.60 wt.%, for *d* series). Although this increase in the particle size is small, it is proportional to the increase in metal loading, suggesting that all samples likely contain a similar number of platinum particles (Figure S2A). This indicates that the synthesis procedure promotes the growth of the platinum particle size with increased metal loading rather than generating new nucleation sites. This is slightly more noticeable in the sequential synthesis. Looking at the HRTEM micrographs, in the case of 0.30(s)-Pt/TiO_2_ and 0.45(s)-Pt/TiO_2_, although platinum shows good dispersion in the sample, there is to some degree more particle agglomeration. This is probably caused by the already present Pt particles acting as nucleation sites, causing the new ones to be preferentially incorporated into them, making the average particle size larger in this series. Moreover, this hypothesis is further confirmed when the Pt particle size (nm) is plotted against the Pt loading (%) (Figure S2B), where the 0.30(s)-Pt/TiO_2_ and 0.45(s)-Pt/TiO_2_ samples have higher Pt contents and particle sizes than their direct method analogs.

**Figure 2:**
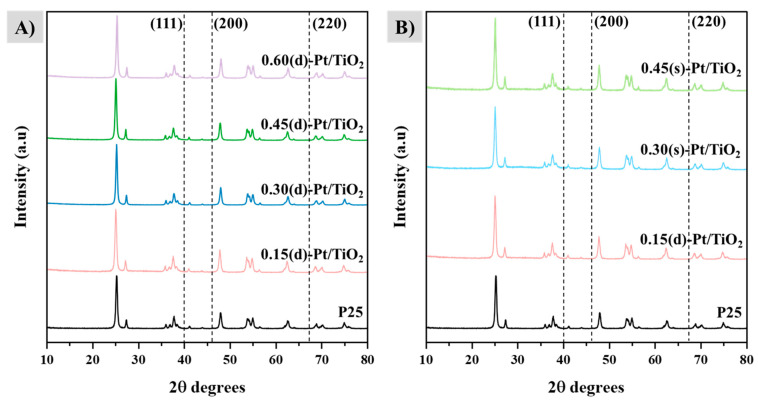
XRD diffractograms of (**A**) direct-series photocatalysts and (**B**) sequential-series photocatalysts. Dashed vertical lines correspond to the expected Pt signals.

The crystallographic structures of the solids were obtained through XRD and diffractograms, and they are shown in Figure 2. Diffractograms of photocatalysts from the direct series are shown in Figure 2A, while Figure 2B shows results from the sequential ones. P25 is also included as a reference.

P25 exhibits the characteristic peaks of anatase, corresponding to signals at 2θ values of 25° (101), 36.9° (103), 38.6° (112), 48.1° (200), 54.1° (105), 55.1° (211), 62.7° (204), 68.7° (116), 70.1° (220), and 75.1° (215); the rutile values are 27.5° (110), 36.2° (101), 41.4° (111), 54.3° (211), 56.5° (220), and 76.7° (202) [31,32]. These same peaks are observed in all photocatalysts, as P25 was used as the support to incorporate platinum. The theoretical signals ascribed to the crystallographic phase of platinum are marked with dashed lines at the positions in the (111), (200), and (220) planes [33], corresponding to signals at 39.6°, 47.4°, and 67.1° [34]. The absence of peaks in these positions is attributed to the small percentage of platinum incorporated through photodeposition as well as to the small particle size, which makes it difficult to identify the metal through this technique. This is in agreement with what was observed through HRTEM: there are not many differences between both synthetic methods, suggesting that platinum is quite homogenously distributed, not forming big agglomerations, as they might have been identified in the diffractograms.

**Figure 3:**
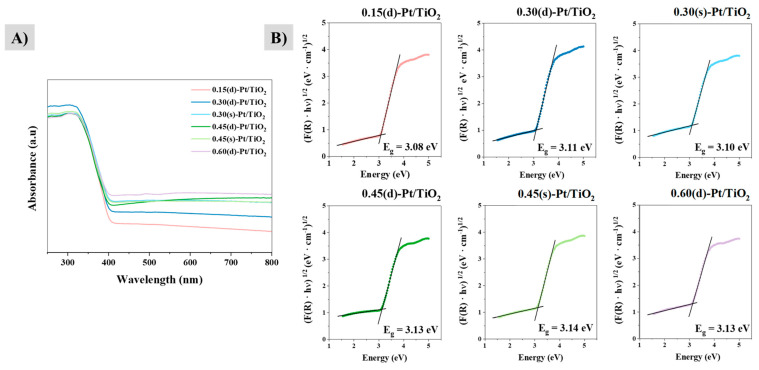
UV-Vis spectroscopy results of the samples. (**A**) Absorbance as a function of wavelength for these systems. (**B**) Bandgap determination from the modified version of the Kubelka–Munk function.

Finally, the UV-Vis absorption spectroscopy results of the six photocatalysts are shown in Figure 3.

The bandgap of P25 is in the range of 3.0–3.2 eV [35,36]. All Pt/TiO_2_ systems present a similar bandgap value to that of P25, in between the 3.08–3.14 eV range (Figure 3B). This parameter has not been modified with respect to the one of P25, and therefore, they would still be active under UV radiation. This is due to the fact that platinum is incorporated on the surface of the photocatalyst through photodeposition, but it cannot be considered a dopant, as it is not included inside the structure of the photocatalyst.

The absorbance spectra of all the photocatalysts are also shown in Figure 3A. The 0.60(d)-Pt/TiO_2_ solid has the highest absorbance in the visible range and 0.15(d)-Pt/TiO_2_ has the lowest. This agrees with the color of the synthesized photocatalyst itself and is caused by the different platinum content: the higher the platinum content, the higher the absorption in the visible spectrum.

### 3.2. Photocatalytic Hydrogen Production Through Glycerol Photoreforming

**Figure 4:**
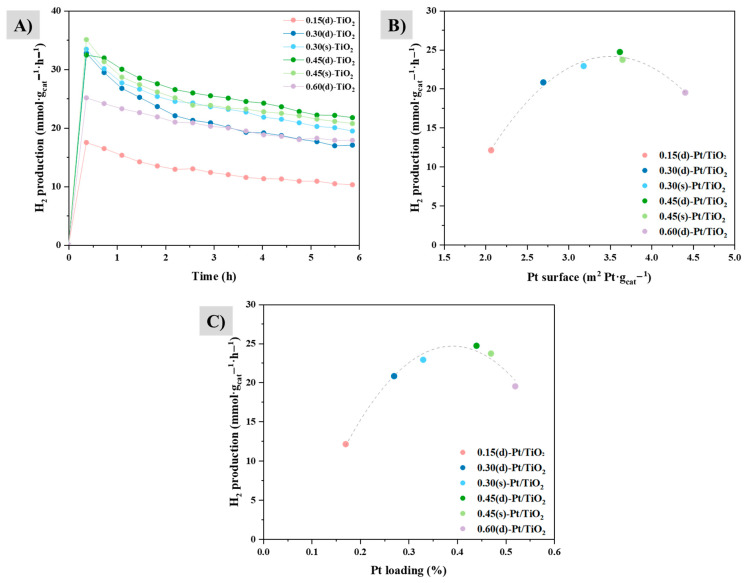
Photocatalytic hydrogen production results from glycerol photoreforming reactions carried out under UV radiation for 6 h. (**A**) The hydrogen production profile curve of semiconductors expressed in millimoles of H_2_ per gram of catalyst and hour. (**B**) The average hydrogen production expressed in millimoles of H_2_ per gram of catalyst as a function of the exposed surface area of platinum. (**C**) The average hydrogen production expressed in millimoles of H_2_ per gram of catalyst as a function of metal loading.

Table 2 and Figure 4 show the results obtained for hydrogen production from glycerol photoreforming using UV radiation at 365 nm for 6 h. In order to establish a relationship with the photocatalytic improvement that the incorporation of platinum entails, bare P25 was tested under the same conditions. Reactions for each Pt/TiO_2_ system were performed in triplicate, so the table and plot show the average value of the results obtained. In all cases, hydrogen production by the photoreforming of glycerol is significantly improved with the addition of platinum, since the production value of bare P25 is only around 2 mmol·g_cat_^−1^·h^−1^ (ca. 6–12 times lower).

The efficiency was first studied with the photocatalysts synthesized through the direct method, adding the appropriate metal precursor volume so as to obtain the desired metal loading. As can be seen in Figure 4A, the 0.60(d)-Pt/TiO_2_ photocatalyst exhibits a lower hydrogen production than the 0.45(d)-Pt/TiO_2_ one (19.5 vs 24.7 mmol·g_cat_^−1^·h^−1^, respectively). Due to this fact, it was decided not to synthesize the 0.60 wt.% Pt catalyst using the sequential method, since according to the catalytic results, the optimum Pt loading does not apply to this metal content. Therefore, this will imply reagent, energy, and time savings regarding the synthesis of materials. Also, 0.15(d)-Pt/TiO_2_ exhibits the lowest production rate; therefore, 0.15 wt.% Pt is far from the optimum metal loading. 

The exposed platinum surface area on the catalyst was calculated as a function of the actual metal loading determined by ICP-MS and by taking into account the amount of catalyst used in each reaction. Figure S3 shows that increasing the Pt metal loading, apart from increasing platinum particle size, results in a rise in the exposed Pt surface area. However, this does not correlate with their hydrogen production activity (Figure 4B), as 0.60(d)-Pt/TiO_2_ is one with the highest surface and exhibits lower gas production than most of the other systems. This again supports the conclusion that 0.60 wt.% Pt metal loading is not the optimum amount because, although it has the largest platinum surface area available to carry out the proton reduction reaction into H_2_, it is likely that higher contents of platinum act as electron–hole pair recombination centers, therefore decreasing the photocatalytic efficiency of the catalyst [37]. Also, it is possible that, as it has a higher platinum content, there are more internal particles that are not able to carry out the reduction process.

Taking this plot (Figure 4B) into account and the one shown in Figure 4C, it is possible to determine that the optimum platinum metal loading for hydrogen production under these conditions is around 0.40 wt.%. As for the difference between the direct and sequential incorporation methods, for the same platinum content, it seems that, in the latter case, the fact that platinum particles tend to incorporate on or in the vicinity of platinum particles incorporated in previous steps (evidenced by HRTEM) is detrimental to the activity (as expressed as mmol H_2_·m^2^_Pt_^−1^·h^−1^) as compared to the direct method, where platinum is slightly more homogeneously distributed on titania (compare Table 2, column 5, entries 3 vs 4 and 5 vs 6). Moreover, incorporating the desired amount of metal in one step is more beneficial in terms of saving energy and time, which makes this synthetic approach preferable to the sequential one. The theoretical H_2_/CO_2_ ratio in the glycerol photoreforming is 2.33. As commented previously, in practice, this ratio is not obtained and, in fact, is higher as a consequence of the accumulation of oxidized intermediates. Results for this parameter are shown in Table 2. The H_2_/CO_2_ ratio could not be determined for P25, as its low H_2_ production evidenced the low yield in glycerol photoreforming under these conditions, and it was impossible to measure the CO_2_ levels, as they were below the detection limit. For the different Pt/TiO_2_ systems synthesized in this work, H_2_/CO_2_ ratios between 7–10 have been obtained, and they were lower for the 0.30(d)-Pt/TiO_2_ solid.

The study of the reaction mechanism is beyond the scope of the present paper, which is focused on the comparison of the Pt/TiO_2_ solids in terms of hydrogen production. Nevertheless, some previous papers of our research group have dealt with this. Therefore, for instance, in Escamilla-Mejía et al. [38], different glycerol initial concentrations were tested, and we found that to reach the theoretical H_2_/CO_2_ ratio value in glycerol photoreforming, the glycerol in water should be around 0.01–0.1% (*v*/*v*). As for the reaction mechanism, glycerol photoreforming on M/TiO_2_ systems seems to proceed through three main parallel routes involving initial oxidation of either C1 or C2 alcohols (forming glyceraldehyde or dihydroxyacetone, respectively) or the C-C cleavage yielding glycolaldehyde [39,40]. Some studies have focused on the possibility of simultaneously producing several added-value products in the liquid phase (e.g., dihydroxyacetone, glyceraldehyde) and hydrogen in the gas phase during glycerol photocatalytic transformation [41,42]. In this sense, a key factor is the relative adsorption strength of the different intermediates on the semiconductor of choice. Therefore, for instance, titania was found to favor the total oxidation of glycerol, whereas WO_3_ led to a high selectivity to glyceraldehyde, which was attributed to the enhanced acidity in WO_3_, which selectively activates C-O bonds in glycerol and facilitates glyceraldehyde desorption [41]. In a more recent study, the production of glyceraldehyde and dihydroxyacetone was further increased through the combination of photo- and electrocatalysis [42].

Although it is difficult to compare hydrogen production rates from different reaction systems reported in the literature, as there are many factors that influence this value (light source power, metal incorporation method, sacrificial agent, etc.), Table 3 shows hydrogen production results from other works, expressed in mmol·g_cat_^−1^·h^−1^, compared to those obtained for the most efficient catalyst in this piece of research. Focusing on results described for the same sacrificial agent (glycerol), our solid produces about three times more hydrogen than another Pt-TiO_2_ system using the same wavelength during irradiation and a platinum loading of 2.1 wt.% versus our 0.45 wt.% incorporated through photodeposition as well [26]. Sola et al. [43] reported some Pt-TiO_2_ systems with a 0.30 wt.% Pt metal loading that produced 42.9 mmol·g_cat_^−1^·h^−1^. However, they tested their efficiency in hydrogen production using ethanol as the sacrificial agent, which as it is a monoalcohol, has less tight adsorption on the catalyst and, therefore, produces hydrogen more easily [44].

## 4. Conclusions

Six photocatalysts were synthesized, incorporating platinum through photodeposition in a nominal content between 0.15 and 0.60 wt.% on TiO_2_. They were subsequently tested on hydrogen production reactions from the photoreforming of glycerol, a very abundant by-product (10 wt.%) in the production of biodiesel. Pt/TiO_2_ systems were synthesized in two different ways: *directly*, adding the appropriate volume of metal precursor so as to obtain the desired metal loading, or *sequentially*, increasing the metal loading of the solid by adding a constant volume of metal precursor in consecutive photodeposition reactions.

The incorporation of the noble metal in titania, studied by ICP-MS, was higher than 85% in all cases, showing that photodeposition is an efficient procedure to incorporate metals on the surface of semiconductors. The images obtained by HRTEM exhibit, on the one hand, that platinum is homogeneously distributed in all catalysts, being slightly more agglomerated on the systems synthesized in several steps. Platinum particles that were already incorporated on the surface of P25, acting as nucleation centers, may account for this. On the other hand, both the direct and sequential incorporation of platinum by photodeposition on TiO_2_ lead to similar metal particle sizes, between 2.3 and 3.6 nm, but it has been shown that the particle size increases with metal loading. Based on the oxidation state of platinum observed by XPS, all the samples present a high percentage of Pt^0^, as a consequence of the UV radiation used during photodeposition.

The results of hydrogen production, expressed in mmol H_2_·g_cat_^−1^·h^−1^ as a function of the platinum surface and metal loading, show that, under our experimental conditions, the optimum metal loading is around 0.40 wt. %, and the 0.45(d)-Pt/TiO_2_ and 0.45(s)-Pt/TiO_2_ systems are the most efficient ones (24.7 and 23.7 mmol H_2_·g_cat_^−1^·h^−1^, respectively). Also, taking into account that incorporating metal in several steps does not imply a benefit in hydrogen production in comparison to incorporating it in one step, carrying out photodeposition in one step is more coherent regarding energy and time savings.

## Figures and Tables

**Table 1:** A summary of the characterization results.

	ICP-MS	XPS				HRTEM	UV-Vis
Catalyst	Pt wt.%	Surface Pt wt.%	Pt^0^ (eV, (At. %))	Pt^2+^ (eV, (At. %))	Pt^4+^ (eV, (At. %))	Pt Size (nm)	Bandgap (eV)
0.15(d)-Pt/TiO_2_	0.17	ND *	ND	ND	ND	2.3	3.08
0.30(d)-Pt/TiO_2_	0.27	0.41	70.8 (81.5)	72.1 (18.5)	ND	2.8	3.11
0.30(s)-Pt/TiO_2_	0.33	0.36	70.7 (81.2)	72.1 (18.8)	ND	2.9	3.10
0.45(d)-Pt/TiO_2_	0.44	0.48	70.8 (66.1)	71.8 (24.3)	73.5 (9.6)	3.4	3.13
0.45(s)-Pt/TiO_2_	0.47	0.56	70.7 (66.1)	72.1 (16.3)	73.7 (17.6)	3.6	3.14
0.60(d)-Pt/TiO_2_	0.52	0.78	70.7 (67.0)	71.9 (8.7)	73.7 (24.3)	3.3	3.13

* ND: not detected.

**Table 2:** Photocatalytic results in glycerol photoreforming reactions under UV radiation.

Catalyst	Pt Surface (m^2^ Pt·g_cat_^−1^)	Hydrogen Production (mmol·g_cat_^−1^·h^−1^)	Hydrogen Production (mol·g_Pt_^−1^·h^−1^)	Hydrogen Production (mmol·m^2^_Pt_^−1^·h^−1^)	H_2_/CO_2_ Ratio
P25	-	1.9	-	-	-
0.15(d)-Pt/TiO_2_	2.1	12.1	7.1	5.9	9.8
0.30(d)-Pt/TiO_2_	2.7	20.8	7.7	7.7	7.5
0.30(s)-Pt/TiO_2_	3.2	22.9	6.9	7.2	7.9
0.45(d)-Pt/TiO_2_	3.6	24.7	5.6	6.8	8.2
0.45(s)-Pt/TiO_2_	3.7	23.7	5.0	6.5	8.0
0.60(d)-Pt/TiO_2_	4.4	19.5	3.7	4.4	8.7

**Table 3:** A comparison of the results of the photocatalytic production of hydrogen obtained in this work, with other results reported in the literature for similar Pt/TiO_2_ systems.

Catalyst	wt.% Pt	Pt Incorporation Method *	Catalyst Loading (g·L^−1^)	Sacrificial Agent	Light Source	H_2_ Rate (mmol·g_cat_^−1·^h^−1^)
TiO_2_ microemulsion	1.50	DP IWI	0.50	Glycerol	300 W Xe lamp	3.68 3.06 [45]
P25	1.00	IP	0.50	Glycerol	30 W LED light (λ = 380 nm)	1.35 [46]
TiO_2_ hydrothermal	0.20	Reaction medium	0.63	Glycerol	150 W Xe lamp	1.25 [47]
Fluorinated hydrothermal TiO_2_	0.50	PD	1.50	Glycerol	8 W UV lamp (λ_max_ = 365 nm)	2.17 [25]
Sol-gel TiO_2_	2.10	PD	1.00	Glycerol	15 W fluorescent tubes (λ_max_ = 365 nm)	7.90 [26]
P25	1.50	IP DP	0.50	Glycerol	300 W Xe lamp	6.5 10.2 [48]
TiO_2_ Sigma Aldrich, St. Louis, MO, USA	0.30	IWI	2.00	Ethanol	175 W Hg lamp	42.9 [43]
P25	2.00	PD	2.00	Methanol	300 W Hg lamp	16.4 [49]
P25	0.50	PCD	2.00	Cellulose	Solar box 1500 e	0.13 [50]
TiO_2_ Sigma Aldrich	0.16	IWI	0.75	Cellulose	8 W UV-A lamp (λ_max_ = 365 nm)	0.13 [51]
TiO_2_	0.16	IWI	0.75	Glucose	8 W UV-A lamp (λ_max_ = 365 nm)	0.18 [52]
P25	0.45	PD	1.00	Glycerol	1.7 W LED light (λ = 365 nm)	24.7 ^a^

* DP: deposition–precipitation; IWI: incipient wetness impregnation: IP: impregnation: PD: photodeposition; PCD: photochemical deposition. ^a^ This work.

## Data Availability

Data will be available on request.

## Supplementary Materials

The following supporting information can be downloaded at: https://www.mdpi.com/article/10.3390/ma17205109/s1, Figure S1: XPS spectrum in Pt4f region of all semiconductors synthesized in this work.; Figure S2: (A) Number of platinum particles present in the catalyst according to its experimentally determined metal loading. (B) Platinum average particle size as a function of metal loading as determined by ICP-MS.; Figure S3: Exposed platinum surface area in each catalyst according to its platinum metal loading.
