# Supplementary material for: Pt-TiO2 Systems for Enhanced Hydrogen Production from Glycerol: Direct vs Sequential Incorporation Through Photodeposition
Source: Materials (Basel). 2024 Oct 19;17(20):5109. doi: 10.3390/ma17205109 (PMC11509525; doi:10.3390/ma17205109)
Supplement: Supplementary file 1 [file materials-17-05109-s001.zip › materials-3242887-supplementary.pdf]

Supplementary Materials

Pt-TiO_2 systems for enhanced hydrogen production from glycerol: direct vs sequential incorporation through photodeposition

Ana M. Carozo [1], Francisco J. López-Tenllado[1], M. Carmen Herrera-Beurnio[1,*], Jesús Hidalgo-Carrillo[1], Juan Martín-Gómez[1], Rafael Estevez[1], Alejandro Ariza-Pérez[1], Francisco J. Urbano[1], Alberto Marinas [1,*]

Departamento de Química Orgánica, Instituto Químico para la Energía y el Medioambiente (IQUEMA), Universidad de Córdoba, E-14071 Córdoba, Spain

* Correspondence: b52hebem@uco.es (M.C.H.-B.); alberto.marinas@uco.es (A.M.)

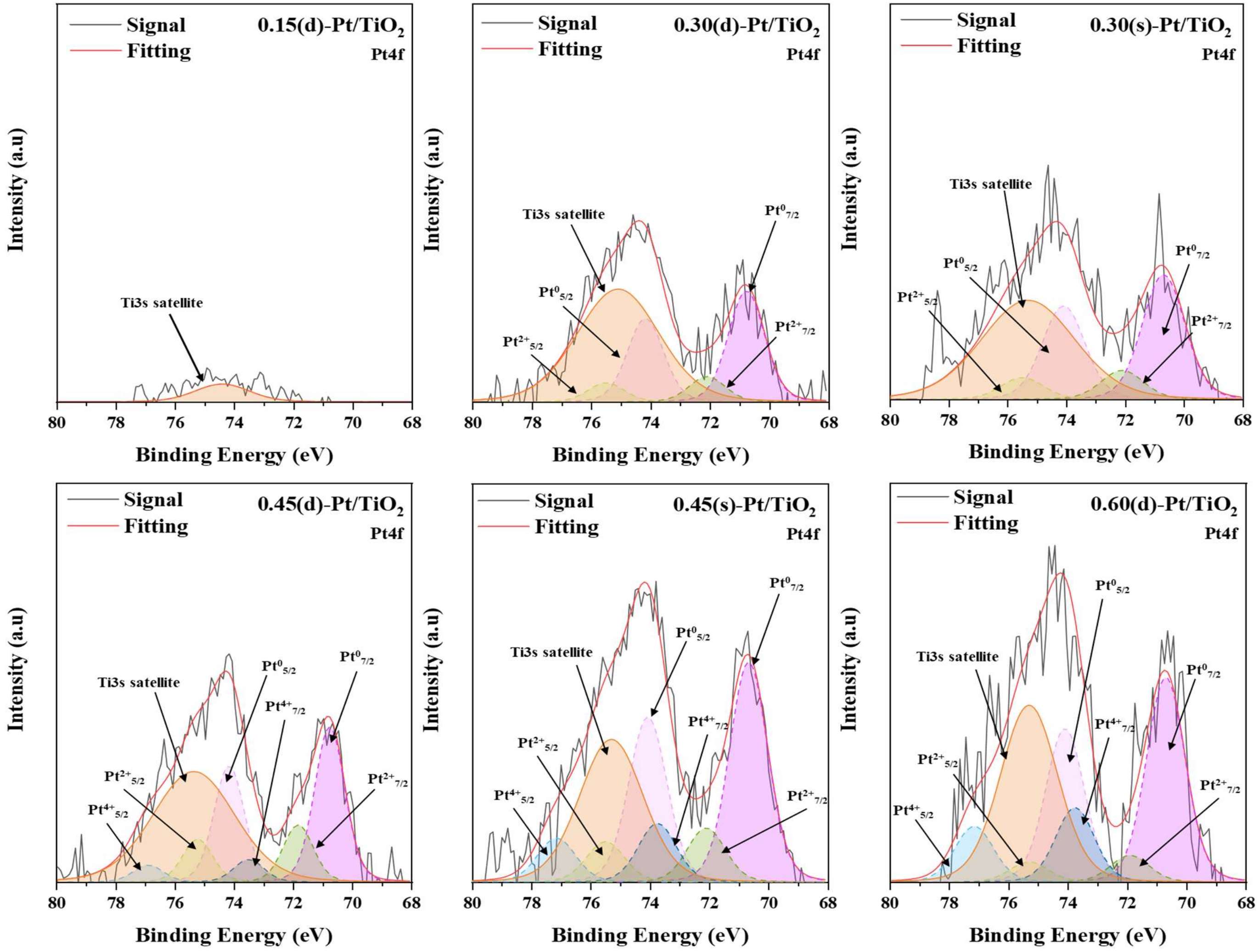

Figure S1. XPS spectrum in Pt4f region of all semiconductors synthesized in this work.

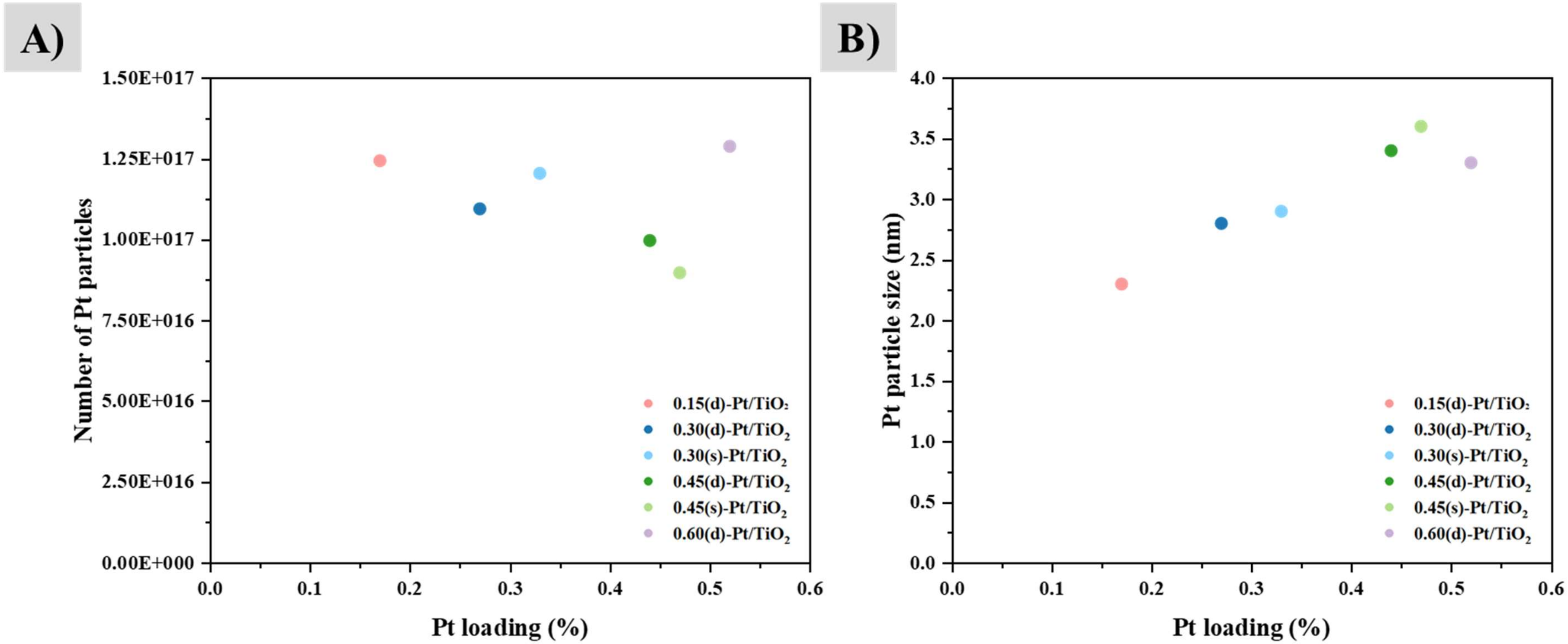

Figure S2. A) Number of platinum particles present in the catalyst according to its experimentally determined metal loading. **B)** Platinum average particle size as a function of metal loading as determined by ICP-MS.

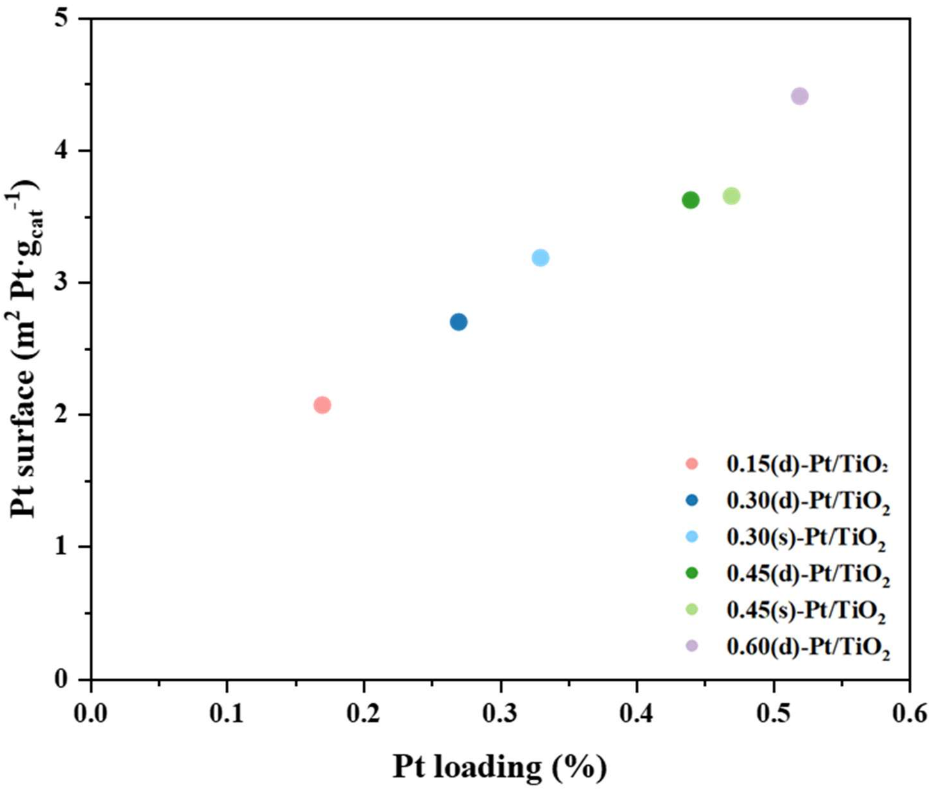

Figure S3. Exposed platinum surface area in each catalyst according to its platinum metal loading.
